# Effects of saffron (*Crocus sativus*) petal ethanolic extract on hematology, antibody response, and spleen histology in rats

**Published:** 2014

**Authors:** Atefeh Babaei, Javad Arshami, Alireza Haghparast, Mohsen Danesh Mesgaran

**Affiliations:** 1 *Department of Animal Sciences, Agriculture Faculty, Ferdowsi University of Mashhad, I. R. Iran*; 2 *Department of Pathobiology, Faculty of Veterinary Medicine, Ferdowsi University of Mashhad, Mashhad, I. R. Iran*

**Keywords:** *Hematology*, *Immune system*, *Rat*, *Saffron petal extract*

## Abstract

**Objective:** Saffron petal is a by-product that contains flavonoids and anthocyanins. In order to study the effects of saffron petal extract (SPE) on blood parameters, immune system, and spleen histology, five treatments (n=6) were used in a completely randomized design.

**Materials and Methods:** The treatments were 0, 75, 150, 225, and 450 mg/kg body weight of SPE. The SPE was injected intraperitoneally to 30 rats (10-week old, weighing 225±15 g) for 14 days. Immunization was performed using 1×10^8^ sheep red blood cells (SRBC) on days 0 and 7 subcutaneously in all treatment groups. On day 15, blood was collected from the heart of rats after anesthesia. One part of samples were poured in heparinized tubes for counting whole blood cells (CBC) and different white blood cells (WBC) and the other part was used to measure IgG using ELISA technique. The spleen was stained by hematoxylin- eosin for histological study. The data were statistically analyzed using ANOVA program and the means evaluation was done using Tukey’s test. Results are presented as mean±SD.

**Results:** Results showed no significant difference between treatments and control group regarding the amount of RBC, HGB, HCT, and PLT. The level of IgG at 75 mg/kg was significantly increased in comparison with other groups. No changes were observed in spleen histology.

**Conclusion:** The results indicate that use of SPE at dose of 75 mg/kg causes an increase in antibody response without any change in hematological parameters and spleen histology.

## Introduction

*Crocus sativus *L. (C. sativus) commonly known as saffron, is a perennial stemless herb of the *Iridaceae* family. It is widely cultivated in Iran and Spain and at a lower scale in other countries, such as Italy, India, and France. Chemical studies of *C. sativus* have shown the presence of constituent such as crocin, croetin, safranal and picrocrocin      (Abolhasani et al., 2005 ▶)  Modern pharmacological extracts of saffron have antitumor )             Abdullaev et al., 2004 ▶; Abdullaev 2001 ▶; Das et al., 2004 ▶) , antioxidant )         Ranmadan et al., 2010 ▶; Assimopoulou et al., 2005 ▶; Sanchez -vioque et al., 2010; Goli et al., 2010 ▶) , antinociceptive and anti-inflammatory    (Hossienzadeh et al., 2002 ▶) , antidepressant      (Karimi et al., 2001 ▶; Akhondzadeh Basti et al., 2007 ▶; Hossienzadeh et al., 2004 ▶) , hypolipaemic (Sheng et al., 2006 ▶), and immunostimulant (Kianbakht et al., 2011 ▶; Sarang et al., 2010 ▶) effects. 

The immune system is involved in the etiology and pathology of many diseases. Modulation of immune responses improves controlling diseases. The subchronic daily use of 100 mg saffron has temporary immunemodulatory activities (Kianbakht et al., 2011 ▶). Saffron petals consist of anthocyanins, glycosides, and flavonoids      (Gil et al., 2002 ▶).  Kaempferol glycoside is the major flavonols (84.0% of total flavonol content) in C. sativus (Goupy et al, 2013). Modern pharmacological studies have shown that saffron petal extract has antitumor (Hosseinzadeh, et al, 2008), antioxidant (Tajali et al., 2008 ▶), antinociceptive and anti-inflammatory    (Hosseinzadeh, et al, 2002 ▶) , and antidepressant       (Akhondzadeh Basti et al., 2007 ▶; Hossienzadeh et al., 2007 ▶)  effects. So far, effects of alcoholic extract of saffron petals on immune system have not been reported. The aim of the present study was to determine the effects of *C. sativus* petals extract on hematology, antibody response, and spleen histology in rats.

## Materials and Methods

**Preparation of extract**

*C. sativus* petals were collected (October 2010) from Torbat-e Heydarieh (khorasan razavi, north east of Iran). A voucher specimen of plant is preserved in the Ferdowsi University of Mashhad Herbarium (FUMH: 31832). Extract of petal powder was prepared using maceration with ethanol (80%, v/v) for 3 days and subsequently, the mixture was filtered and concentrated under reduced pressure at 35 C. Finally, the extracts were dissolved in normal saline       (Hossienzadeh et al., 2002 ▶;  Akhondzadeh Basti et al., 2007 ▶)  .

**Photochemical Screening**

Photochemical screening of the extract was performed using the following regents and chemicals: Alkaloids with Mayer regent, flovonoids using Mg and HCL, anthocyanins using HCL, tannins with ferric chloride, and saponins with ability to produce suds      (Evan, 1996)     (Trease GE et al., 1983 ▶) .

**Animals**

Wistar rats 10-week-old and weighing 225±15±15 g obtained from Pasteur Institute of Iran. These animals were maintained in animal house facility of Ferdowsi University of Mashhad. They were housed in individual cages under standard laboratory conditions in 12h/12h light/dark cycle and at a temperature of 21-23 C. Rats had free access to food and water. According to ethical regulations on animal research, all of these animals received proper human cares. Thirty rats in five treatment groups (n=6) were used in a completely randomized design. Control group received normal saline (10 ml/kg body weight) and treatment groups received 75,150, 220, and 450 mg/kg body weight of SPE. The SPE were injected intraperitoneally to rats for 14 days.

**Blood sampling and hematological analysis**

On day 15, rats were anesthetized and blood was collected from the heart and white blood cell count (WBC), red blood cell count (RBC), percent hemoglobin (HGB), hematocrit (HCT), mean cell hemoglobin (MCH) and mean cell hemoglobin concentration (MCHC), mean corpuscular volume (MCV), and platelet count were measured using cell counter (Celltacα -NIHON KOHDEN- GERMANY). For differential count of white blood cells, the smear slides stained with Giemsa was prepared and lymphocyte, monocyte, and neutrophil percentages were counted using an optical microscope.

**Humoral antibody response to SRBC**

Groups of five rats, each were immunized by injecting 0.5 ml of 1×10^8^ SRBC/ml intrapeitoneally (i.p.) on day 0 and challenged on day 7 by injecting an equal volume of SRBC (i.p.) (Vaibhav et al., 2010 ▶; Agrawal et al., 2010). Blood samples were collected on day 14 for IgG antibody assay. IgG level was measured in serum samples using the rat IgG ELISA (enzyme linked immune sorbent assay) kit (GWB- BAA3A8, Genway-Biotech, USA).

**Spleen histology**

The spleen was weighed using an electronic balance (Sartorius, TE214 S) and the spleen index was calculated according to the following formula (Xia et al., 2010 ▶):

Spleen index=spleen weight (g)/ body weight (g). After the spleen was removed and weighed, it was washed by normal saline, cut into five Mm sections and placed in 10% formalin. After 3 days, sections with a thickness of 5 micrometer were prepared and stained by hematoxylin-eosin for histological study (Mc Manus, 1948 ▶).

**Statistical analysis**

Data were analyzed using SAS 9.1 software and the data were statistically analyzed using ANOVA program and the means evaluation was done using Tukey’s test. A value of p<0.05 was considered as statistically significant. Results are presented as mean±SD.

## Results

**Photochemical screening**

Photochemical analysis of *C. Sativus* petal extract showed that the extract contains flovonoids, anthocyanins and tannins and does not contain alkaloids and saponins.

**Hematological analysis**

As shown in Table 1, the number of white blood cells was significantly increased as compared with control group (p<0.05). There was no significant difference regarding RBC count, hemoglobin, hematocrit percentage, mean corpuscular volume, mean cell hemoglobin, and mean cell hemoglobin concentration between the control group and groups treated with extract of saffron petals.

Percentage of lymphocytes, neutrophils and monocytes count between the control group and groups treated with SPE showed no significant difference (Table 2).

**Spleen histology**

**Figure 1:**
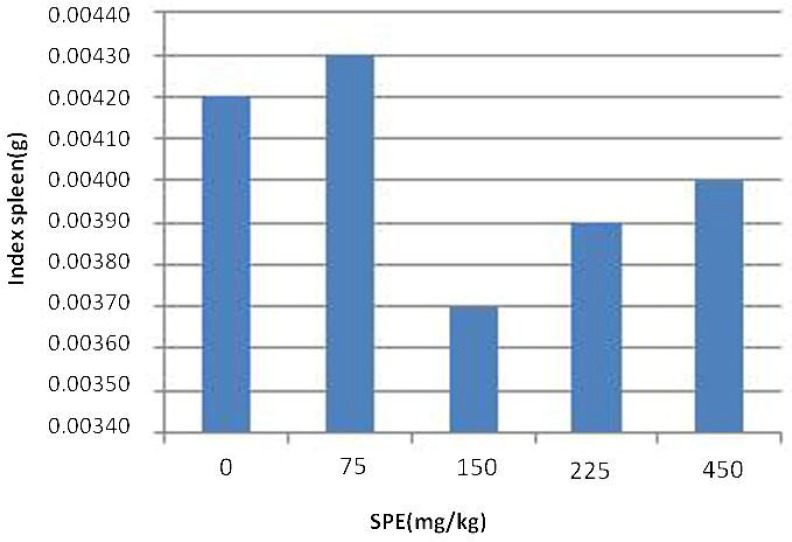
The effect of SPE on spleen index in rats. Values are expressed as mean±SD

**Figure 2:**
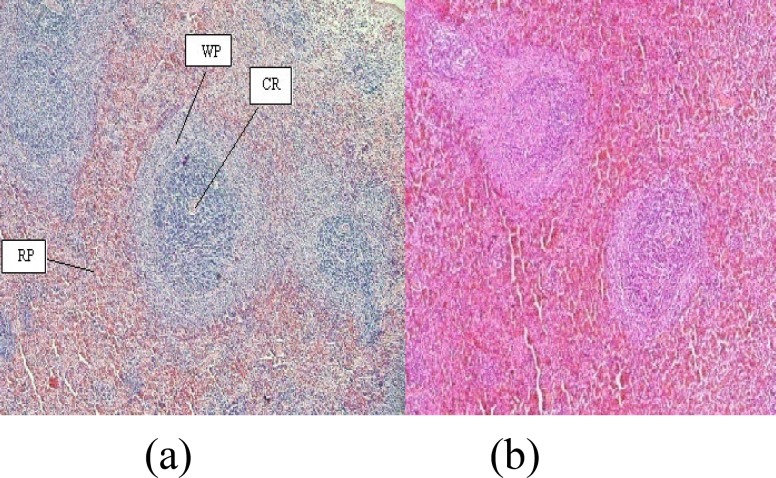
Transected spleen tissue controls (a) and 450 (mg/kg) SPE groups. RP: red pulp, WP: white pulp, CA: central artery. Magnification ×100

Studies of spleen index showed no significant difference between treatment groups and control group (Figure 1). The histology of spleen was observed to remain unchanged following exposure to extract of saffron petal at the doses of 75, 150, 225, and 450 mg/kg body weight. The morphology of splenocytes in all treatment groups was not different from control group (Figure 2).

**Humoral antibody response to SRBC**

**Figure 3:**
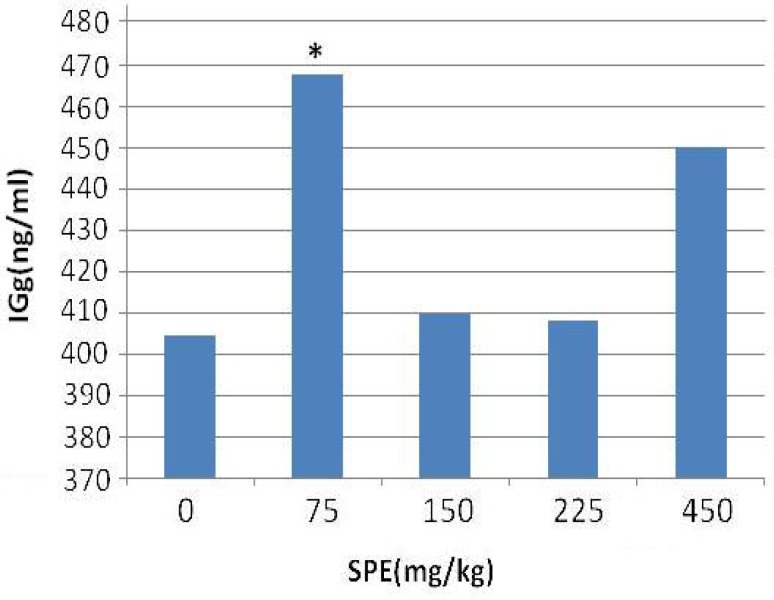
The effect of SPE on IgG concentration in rats**. **Values are expressed as mean±SD. SPE: saffron petals extract, IgG: Immunoglobulin G. *significantly different from the control (p<0.05).

Concentration of IgG in the treatment groups increased as compared with the control group. This increased level of IgG was significant at the dose of 75 (mg/kg) (p<0.05) (Figure 3).

**Table 1:** The effect of SPE on hematological parameters in rats

**Parameters**	**Treatment**				
	**0 (mg/kg)**	**75 (mg/kg)**	**150 (mg/kg)**	**225 (mg/kg)**	**450 (mg/kg)**
**WBC (10** ^3^ **/μl)**	3.00±1.09	4.06±2.28	6.10±1.30 *	5.03±0.72	7.12±2.00 *
**RBC (10** ^6^ **/ μl)**	7.46±0.55	7.59±0.33	7.43±0.53	7.82±0.49	8.04±0.90
**HGB ** **(gm/dl)**	15.27±1.34	15.05±0.50	14.90±1.09	15.78±0.50	15.82±1.52
**HCT (%)**	41.42±3.04	40.67±2.10	39.37±2.89	42.02±1.42	42.65±3.97
**MCH ** **(pg)**	20.45±0.54	19.82±0.35	20.07±0.06	20.23±1.20	19.72±0.89
**MCHC ** **(gm/dl)**	36.83±1.04	37.02±0.78	37.87±0.68	37.57±1.08	37.08±0.44
**MCV** ** (fl)**	55.55±0.76	53.57±1.03	53.02±1.43	53.82±1.85	53.17±2.18

Values are expressed as mean±SD, n=6, SPE: saffron petals extract, WBC: white blood cell count, RBC: red blood cell count, HGB: percent hemoglobin, HCT: hematocrit, MCH: mean cell hemoglobin, MCHC: mean cell hemoglobin concentration, and MCV: mean corpuscular volume as compared with group 0 (control).

* significantly different from the control, (p<0.05).

**Table 2:** The effect of SPE on differential leukocyte count in rats

**Parameters**	**Treatment**				
	**0 (mg/kg)**	**75 (mg/kg)**	**150 (mg/kg)**	**225 (mg/kg)**	**450 (mg/kg)**
**Lymphocytes (%)**	67.00±2.65	74.00±1.73	77.00±10.15	71.33±2.08	68.00±5.57
**Neutrophil (%)**	27.67±1.53	22.00±1.00	22.00±5.20	23.33±3.06	28.33±5.69
**Monocytes (%)**	5.33±1.15	4.00±1.00	4.33±1.53	5.33±1.53	3.67±0.58

## Discussion

Many herbal preparations alter immune function and display an array of immunomodulatory effects (Sarang et al., 2010 ▶). In various *in vitro* and *in vivo* studies, herbal medicines have been reported to modulate cytokine secretion, histamine release, immunoglobulin secretion, cellular co-receptor expression, lymphocyte activation, and phagocytosis (Patwadhan et al., 2005 ▶; Plaeger, 2003 ▶). However, their clinical efficacy is limited and associated with complications (Wieland et al., 2005 ▶). In the present study, for the first time, the effects of SPE on hematological parameters, humoral immune system, and histology of spleen was evaluated in rats.

The results obtained from this study indicated that use of saffron petals at a dose of 75 mg/kg has a clear immunostimulatory effect as it was shown by the significant increase in IgG concentration. The results of our study indicated that use of saffron petals at the doses of 75, 150, 225, and 450 mg/kg has no effect on hematological parameters. In anemia, hemolysis and decreased production of red blood cells are effective. Here, in the blood-extracted plasma, hemolysis was not observed and no significant changes in blood cell count (RBC), percent hemoglobin (HGB), hematocrit (HCT), and mean corpuscular volume (MCV) were observed. In general, we can conclude that the injected dose did not cause any type of anemsreia. 

It was shown previously that subacute toxicity of saffron petal extract in rats, regarding the amount of RBC, HGB and HCT was significantly decreased (Karimi et al., 2004 ▶). Our results showed that, use of saffron petals causes an increase in the number of white blood cells in treatment groups and also control group which indicates immunemodulatory activities of saffron petal. Lymphocytes, neutrophils and monocytes count showed no significant difference (p>0.05) between the control and treated groups with different doses of extract of saffron petals. In evaluation of the effect of saffron on immune system in healthy individuals, monocyte percentages decreased significantly which can affect innate and humoral immune response (Ranmadan et al., 2010 ▶). 

Spleen is one of the principle sites for the initiation of most primary immune responses for B lymphocyte activation and the production of antibodies. Results showed that ethanolic extract of saffron petal have no significant difference in index of spleen. No changes were observed in spleen histology. Saffron petals had not toxic effect in given doses and did not cause spleen tissue damage. As indicated earlier, our results showed that use of saffron petals, increases the serum level of IgG in treatment groups. The results indicated that use of saffron petals at dose of 75 mg/kg causes an increase in antibody response without any change in hematological parameters and spleen histology. 

An important effect of flavonoids is scavenging of oxygen-derived free radicals. *In vitro* experimental systems also showed that flovonoids possess anti-inflammatory, antiallergic, and anticacinigenic properties (Middle et al., 1998 ▶). Positive effects of flavonoids on immune responsiveness might have a variety of underlying mechanisms. Activated immune cells generate free radicals and increase oxidative stress (Costantini, 2006 ▶; Horak et al., 2007 ▶), while T cell and B cell based immune reactions are highly sensitive to oxidative stress(Von Schant et al., 1999 ▶). Role of T helper (Th) cells in the effect of saffron petal should not be disregarded because there is a complex yet intricate network of immune response regulated by the Th cells which can stimulate the B lymphocyte to produce IgG. Thus, saffron probably induces some of these effects by stimulating the secretion of Th cell cytokines such as IFN- which is the main cytokine to stimulate the B lymphocytes for IgG production. Therefore, saffron petal could be used to increase the secondary immune responses (IgG) against pathogens.
